# CTLA-4 Facilitates DNA Damage–Induced Apoptosis by Interacting With PP2A

**DOI:** 10.3389/fcell.2022.728771

**Published:** 2022-02-24

**Authors:** Qiongyu Yan, Bin Zhang, Xi Ling, Bin Zhu, Shenghui Mei, Hua Yang, Dongjie Zhang, Jiping Huo, Zhigang Zhao

**Affiliations:** ^1^ Department of Pharmacy, Beijing Tiantan Hospital, Capital Medical University, Beijing, China; ^2^ Department of Neurosurgery, Beijing Tiantan Hospital, Capital Medical University, Beijing, China

**Keywords:** CTLA-4, PP2A, ATM, DNA damage response, apoptosis

## Abstract

Cytotoxic T-lymphocyte–associated protein 4 (CTLA-4) plays a pivotal role in regulating immune responses. It accumulates in intracellular compartments, translocates to the cell surface, and is rapidly internalized. However, the cytoplasmic function of CTLA-4 remains largely unknown. Here, we describe the role of CTLA-4 as an immunomodulator in the DNA damage response to genotoxic stress. Using isogenic models of murine T cells with either sufficient or deficient CTLA-4 expression and performing a variety of assays, including cell apoptosis, cell cycle, comet, western blotting, co-immunoprecipitation, and immunofluorescence staining analyses, we show that CTLA-4 activates ataxia–telangiectasia mutated (ATM) by binding to the ATM inhibitor protein phosphatase 2A into the cytoplasm of T cells following transient treatment with zeocin, exacerbating the DNA damage response and inducing apoptosis. These findings provide new insights into how T cells maintain their immune function under high-stress conditions, which is clinically important for patients with tumors undergoing immunotherapy combined with chemoradiotherapy.

## Introduction

T lymphocytes (T cells) execute various functions in defense against pathogens and tumors and interact with other cells involved in immune responses. T cells have a life span of several decades and the highest proliferative potential, and T-cell DNA is damaged by diverse endogenous and environmental stress factors (Opzoomer et al., 2019). Therefore, these cells have developed a complex DNA damage response (DDR) to promote the maintenance of genome integrity (Bouwman and Jonkers, 2012). Appropriate communication between cell cycle regulation, DDR, and apoptosis is important to ensure the effectiveness of DDR (Vermeulen et al., 2003; Finn et al., 2012). Double strand breaks (DSBs) are the most harmful DNA lesions in cells. These “dirty” DSBs that remain “uncleaned” may cause gene mutations, genomic instability, and carcinogenesis (Ciccia and Elledge, 2010; Hanahan and Weinberg, 2011). Apoptosis is a major type of cell death in which unwanted cells are removed. Therefore, the identification and development of mechanisms that induce T-cell apoptosis or increase susceptibility to genomic stress are useful for maintaining the health of organisms.

Conventional cancer therapies, including radiotherapy and/or chemotherapy, not only lead to the direct killing of cancer cells but also induce immunogenic cell death. Immune cells undergo apoptosis or lose their function during cancer therapy—problems that urgently require a solution. Immunotherapy, also known as immune checkpoint blockade, is currently one of the most exciting and promising modalities of investigation and development in the field of cancer therapy, and T cells play central roles in cancer immunotherapy (Shklovskaya and Rizos, 2021). Cytotoxic T-lymphocyte–associated protein 4 (CTLA-4) is an immunoregulatory molecule found on T cells and a negative regulator of T-cell activation (Teft et al., 2006). CTLA-4 plays a critical role in maintaining the balance of immune homeostasis, functioning as an immune checkpoint (Schlosser et al., 2016). Since 2000, immune checkpoint inhibitors such as ipilimumab and tremelimumab, CTLA-4 inhibitors that are used in a new and effective treatment strategy for immunotherapy in cancer, have been in clinical development (Chang et al., 2019). The incorporation of chemotherapy, radiotherapy, or chemoradiotherapy with immunotherapy may enhance antitumor immune responses (Chicas-Sett et al., 2018; Wang et al., 2019; Paz-Ares et al., 2021). However, no relevant studies examining the effect of conventional cancer therapies on the immunosuppressive role of CTLA-4 have been published. In one study, CTLA-4–deficient mice were born healthy but exhibited a large number of activated T cells within 5–6 days of birth and died when 18–28 days old as a result of fatal hyperactivation of the immune system and tissue destruction (Sobhani et al., 2021). Based on these results, CTLA-4 has been shown to downregulate T-cell responses and modulate peripheral self-tolerance. Because of this negative regulation, CTLA-4 has been proposed to be related to several autoimmune diseases, although the mechanisms are not fully understood (Ghaderi, 2011). CTLA-4 regulates T-cell activation through multifaceted pathways. Because CTLA-4 and CD28 show significant homology, considerable information is available on CTLA-4 competition with CD28 (Liu et al., 2021). However, very little information is available regarding the inherent plasticity of CTLA-4 signaling. A striking characteristic of CTLA-4 is the conservation of its cytoplasmic domain, implying that this segment is essential for its functions (Qureshi et al., 2012). Some studies have shown that CTLA-4 negatively regulates T-cell activation through the delivery of an inhibitory signal by its cytoplasmic tail (Greenwald et al., 2002; Teft et al., 2009; Haanen and Robert, 2015). Recent studies have shown that CTLA-4 is mainly located in the cytoplasm of resting T cells, while the CTLA-4 level in the cell membrane is higher during T-cell activation, with its membrane level changing in a very dynamic (unstable) manner (Van Coillie et al., 2020). Other reports have documented various CTLA-4 gene expression levels in different cell lines, including T lymphoid, B lymphoid, myeloid, and erythroid (K562)–derived cells; granulocytes; stem cells; and placental fibroblasts (Wang et al., 2002; Pistillo et al., 2003; Contardi et al., 2005). CTLA-4 expression is mainly detected in the cytoplasm of all these cells, except granulocytes. However, the precise function of CTLA-4 in the cytoplasm remains unclear.

Protein phosphatase 2A (PP2A) is a Ser/Thr protein phosphatase that is highly conserved from yeast to humans (Shi, 2009). PP2A consists of three subunits, including a common scaffolding subunit A, two subtypes of catalytic subunit C (α and β), and a regulatory subunit B. PP2A-B was shown to mediate the specificity and localization of PP2A. PP2A-A and PP2A-C comprise the essential enzyme core (Janssens and Goris, 2001). PP2A is a well-known phosphatase that participates in the administration of the DNA damage response (Ramos et al., 2019; Lei et al., 2020). PP2A controls the DNA damage response by regulating the level of ataxia–telangiectasia mutated (ATM) autophosphorylation/activation, which was formerly proven to facilitate DNA damage repair, regulate the cell cycle, and induce apoptosis (Goodarzi et al., 2004). The cytoplasmic CTLA-4 population is associated with PP2A and has been shown to regulate the Raf/MEK/ERK path in T cells (Chuang et al., 2000; Teft et al., 2009). However, the relationship between DDR signaling and the PP2A-CTLA-4 interaction in the cytoplasm has not yet been clarified.

In the present study, we reveal for the first time that CTLA-4 activates ATM by anchoring the ATM inhibitor PP2A in the cytoplasm of T cells following transient treatment with zeocin and, consequently, exaggerating the DNA damage response and inducing apoptosis.

## Materials and Methods

### Mice

B7-1/B7-2^−/−^ and B7-1/B7-2/CTLA-4^−/−^ C57BL/6 mice were purchased from Suzhou Cyagen Co. (Suzhou, Jiangsu, China). About 6- to 8-week-old female mice were used in this study. Experiments on the mice were performed in accordance with approved protocols.

### Cell and Transfection

B7-1/B7-2^−/−^ and B7-1/B7-2/CTLA-4^−/−^ T cells were isolated from the lymph nodes of the indicated mice using CD4 MicroBeads (Miltenyi Biotec, #130117043). CD4^+^ T cells were cultured in a sterile RPMI-1640 medium (Gibco, #A1049101) supplemented with 10% fetal bovine serum (Gibco, #16170078), 1% penicillin/streptomycin (Gibco, #15070063), 10 mM HEPES buffer (Gibco, #15630106), 2 mM L-glutamine, and 50 mM 2-mercaptoethanol (Gibco, #21985023).

HeLa cells were purchased from ATCC and cultured in DMEM (Gibco, #11965084) supplemented with 10% fetal bovine serum. The constructs were transiently transfected into the HeLa cells using Lipofectamine™ 2000 (Invitrogen, #11668019) according to the manufacturer's instructions. About 24 hours after transfection, the cells were treated with 500 μg/ml zeocin (XYbio, #X10014) for 1 h in an incubator, and experiments were subsequently conducted.

The cells were maintained at 37°C in a humidified atmosphere containing 5% CO_2_.

### Plasmid Construction

A plasmid encoding human Myc-CTLA-4 was purchased from OriGene (#NM005214). GFP-CTLA-4 constructs were prepared by excising full-length CTLA-4 from Myc-CTLA-4, and subcloning it into the EcoR I and Kpn I sites of the pEGFP-N1 vector. GFP-CTLA-4-TAIL constructs were prepared by excising the transmembrane plus cytoplasmic tail of CTLA-4 from Myc-CTLA-4 and subcloning this fragment into the EcoR I and Kpn I sites of the pEGFP-N1 vector.

Mutagenesis of Myc-CTLA-4 and GFP-CTLA-4 was performed using a QuikChange Site-Directed Mutagenesis Kit (Agilent, #210518). The sequences of the forward and reverse primers were as follows:

A602T, F: 5′-ggg​ggc​att​ttc​aca​aag​acc​cct​gtt​gta​aga​gg-3′,

R: 5′-cct​ctt​aca​aca​ggg​gtc​ttt​gtg​aaa​atg​ccc​cc-3′, and

A653T, F: 5′-cgc​gta​ttg​atg​gga​ata​aaa​aaa​ggc​tga​aat​tgc​ttt​tca​c-3′,

R: 5′-gtg​aaa​agc​aat​ttc​agc​ctt​ttt​tta​ttc​cca​tca​ata​cgc​g-3′.

### Immunofluorescence Staining

Immunofluorescence staining of HeLa cells was performed as described previously (Yan et al., 2017). Briefly, the cells were cultured on coverslips, washed with PBS, fixed with 4% paraformaldehyde for 10 min at room temperature, permeabilized with 0.2% Triton X-100 for 2 min, and blocked with 1% BSA for 1 h. The cells were then incubated with anti-Myc (Abcam, #ab32072, 1:200) and anti-PP2A-A antibodies (Santa Cruz Biotechnology, #sc-13600, 1:50) at 4°C overnight. Alexa Fluor 594-conjugated goat anti-rabbit IgG (Molecular Probes, #A31632, 1:500) and Alexa Fluor 488-conjugated goat anti-mouse IgG secondary antibodies (Molecular Probes, #A31619, 1:500) were used. The nuclei were detected using DAPI staining. The images were acquired with a laser scanning confocal microscope (Zeiss, LSM 710).

The T cells were washed with PBS and plated on coverslips coated with poly-L-lysine (Sigma, P8130). The next steps were similar to those described in the HeLa cell experiment mentioned above. The primary antibodies used for this experiment were anti-P-ATM (GeneTex, GTX132146, #sc-47739, 1:100), anti-53BP1 (Cell Signaling Technology, #4937, 1:200), and anti-γH2AX (Cell Signaling Technology, #9718, 1:200). The percentage of cells with γH2AX and 53BP1 nuclear foci was quantified using ImageJ software, and the data were plotted using GraphPad Prism software.

### Immunoblotting

Western blotting was performed as previously described (Yan et al., 2017). Briefly, the cells were lysed in a cell lysis buffer for western blotting and immunoprecipitation (IP) (Beyotime, #P0013) containing protease inhibitors (Roche, #04693116001) and phosphatase inhibitors (Coolaber, #SL1087, 1:100), followed by centrifugation at 14,000 *g* for 30 min at 4°C. The supernatant was collected; the protein concentration was estimated; and equal amounts of protein from each sample were separated on SDS-PAGE and electrotransferred to polyvinylidene fluoride membranes. The membranes were blocked with 5% nonfat milk in TBS-T buffer for 1 h at room temperature and incubated with primary antibodies overnight at 4°C. The following primary antibodies were used: DNA Damage Antibody Sampler Kit (Cell Signaling Technology, #9947, 1:1,000), anti-phospho-Histone H2AX (Cell Signaling Technology, #2577, 1:1,000), anti-p53 (Cell Signaling Technology, #2524, 1:1,000), anti-phospho-p95/NBS1 (Cell Signaling Technology, #3001, 1:1,000), anti-MRE11 (Cell Signaling Technology, #4895, 1:1,000), anti-RAD50 (Santa Cruz Biotechnology, #sc-74460, 1:200), anti-NBS1 (Cell Signaling Technology, #3002, 1:1,000), anti-CTLA-4 (Santa Cruz Biotechnology, #sc-376016, 1:50), anti-GFP (Abmart, #7G9, 1:2000), and anti-actin (Sigma, #A5441, 1:1,000). On the second day, the membranes were washed and incubated with the appropriate HRP-conjugated secondary antibodies (Jackson Research, 1:5,000) for 1 h at room temperature. Protein bands were detected using chemiluminescence (ECL) reagent (Millipore, CPS350) and evaluated using densitometry. The experiment was repeated three times independently.

### 2.6 Co-immunoprecipitation

Co-immunoprecipitation (co-IP) was performed as previously described (Yan et al., 2017). The cells were washed once with cold PBS, lysed in lysis buffer for IP (Beyotime, #P0013) containing a protease inhibitor cocktail (Roche) at 4°C for 1 h, and then centrifuged at 12,000 *g* for 15 min at 4°C. After centrifugation, a small fraction of the supernatant was frozen for subsequent analysis (cell extract) as the input. The remaining fraction was incubated at 4°C with 1 μg of the desired antibody on a rotator overnight. Then, the mixtures were incubated with Protein A/G agarose beads (Abcam, #ab193262) with rotation at 4°C for 6 h. After the incubation, the beads were washed three times with wash buffer, boiled in loading buffer at 95°C, and then subjected to western blotting. The following specific secondary antibodies were used to avoid overlap of the bands for the target proteins with those of the immunoglobulin light chain and heavy chain: IPKine HRP Goat AntiMouse IgG HCS (Abbkine, #A25112, 1:5,000), IPKine HRP Goat AntiRabbit IgG HCS (Abbkine, #A25222, 1:5,000), IPKine HRP AffiniPure Goat AntiMouse IgG Light Chain (Abbkine, #A25012, 1:5,000), and IPKine HRP AffiniPure Mouse AntiRabbit IgG Light Chain (Abbkine, #A25022, 1:5,000).

### Comet Assay

A neutral comet assay was performed as previously described (Yan et al., 2017). DNA damage was quantified with a neutral comet assay (CometAssay Kit, Trevigen, #4250–050) according to the manufacturer's instructions. Briefly, after zeocin treatment for 1 h, the B7-1/B7-2^−/−^ and B7-1/B7-2/CTLA-4^−/−^ cells were harvested after the indicated periods, placed in molten agarose, and then spread on the surface of comet slides. After a series of incubations, lysis, and washes at 4°C, the slides were immersed in the lysis solution for electrophoresis, stained with SYBR Green, and then examined with a Zeiss ImagerA2 microscope. The comet assay was quantitated by measuring the percentage of cells with a tail (comet +) from 500 cells using Adobe Photoshop CS6.

### Isolation of Cytoplasmic and Nuclear Cell Fractions

Nuclear extracts were prepared using an NE-PER Nuclear Cytoplasmic Extraction Reagent Kit (Thermo Scientific™, #78835) according to the manufacturer's instructions. Briefly, after stimulation with zeocin, the cells were collected, washed twice with cold PBS, and harvested by centrifugation at 800 rpm for 3 min. The cell pellets were resuspended in cytoplasmic extraction reagent I plus protease inhibitor cocktail by vortexing and allowed to swell on ice for 10 min. Then, cytoplasmic extraction reagent II was added, incubated for 1 min on ice, and the mixture was centrifuged for 5 min at 16,000 *g* at 4°C. The supernatant fraction was obtained as the cytoplasmic extract, transferred to a clean tube, and stored at −80°C. For further nuclear protein extraction, the sediment was suspended in a nuclear extraction reagent by vortexing, incubated on ice for 10 min, and then centrifuged for 10 min at 16,000 *g* at 4°C. The supernatant was obtained as the nuclear extract.

### Cell Apoptosis Assay

Cell apoptosis was analyzed using an Annexin V-FITC/propidium iodide (PI) apoptosis assay kit (Beyotime, #C1062M) according to the manufacturer's instructions. Briefly, the B7-1/B7-2^−/−^ and B7-1/B7-2/CTLA-4^−/−^ T cells were harvested and washed twice with cold PBS. After treatment, the cells were suspended in Annexin V binding buffer containing Annexin V-FITC/PI. After incubation on ice in the dark for 30 min, the cell apoptosis rate was measured by flow cytometry.

### Cell Cycle Analysis

The cell cycle assay was performed using the Cell Cycle and Apoptosis Analysis Kit (Beyotime, #C1052) according to the manufacturer's instructions. Briefly, the HeLa cells were collected and fixed with cold 70% ethanol overnight at 4°C. After centrifugation (1,200 rpm, 5 min, 4°C), the cells were washed with cold PBS. Subsequently, PI was used to stain the cells in the presence of 20 μg/ml RNase A in the dark at room temperature for 30 min. The cell cycle was measured by flow cytometry.

## Results

### CTLA-4 Increases the Risk of T Cells Undergoing DNA Damage–Induced Apoptosis

**FIGURE 1:**
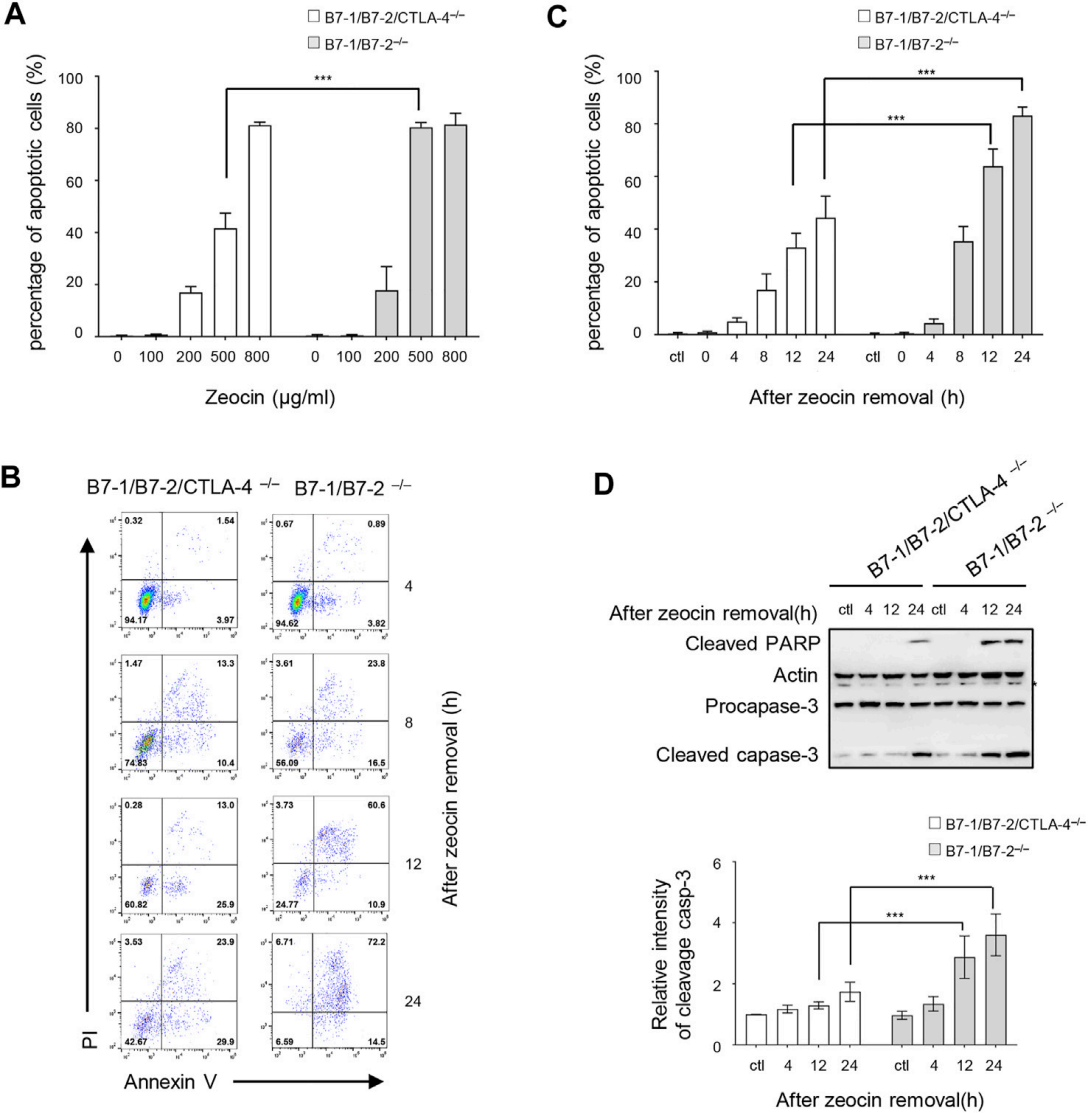
CTLA-4 increases the risk of T cells undergoing DNA damage–induced apoptosis. **(A)** Percentage of apoptotic cells (Annexin V-positive stained cells). CD4^+^ T cells from B7-1/B7-2^−/−^ and B7-1/B7-2/CTLA-4^−/−^ mice were treated with 100, 200, 500, or 800 μg/ml zeocin for 1 h and then cultured without zeocin for 24 h. The cells were collected, and apoptosis was detected. These results are representative of three experiments. All values represent the averages ±SEM of three independent experiments. ***p < 0.001. Ctl indicates untreated control cells. **(B)** Annexin V-PI dual-staining assay and flow cytometry analysis. CD4^+^ T cells from B7-1/B7-2^−/−^ and B7-1/B7-2/CTLA-4^−/−^ mice were treated with 500 μg/ml zeocin for 1 h, cultured without treatment for the indicated times and collected to detect apoptosis. **(C)** Percentage of apoptotic cells (Annexin V-positive stained cells) of the cells shown in **(B)**. These results are representative of three experiments. All values are presented as the averages ±SEM of three independent experiments. ***p < 0.001. **(D)** The levels of cleaved PARP and caspase-3 in CD4^+^ T cells from B7-1/B7-2^−/−^ and B7-1/B7-2/CTLA-4^−/−^ mice were detected using western blotting. The asterisk indicates a nonspecific band. The bar chart shows the fluorescence intensity of the cleaved caspase-3 band in the immunoblot.

We assessed the CTLA-4 activity in response to the genotoxic stress to explore the function of cytoplasmic CTLA-4 in the DDR. Zeocin, a member of the bleomycin family, binds to and intercalates into DNA, leading to the formation of DSBs in a manner similar to that of ionizing radiation (Peres de Oliveira et al., 2020). A previous study verified that zeocin treatment induced a significant decrease in the number of T cells in the peripheral blood (Hu et al., 2018). CTLA-4–deficient mice die at 18–28 days due to the generation of self-reactive T cells (Chambers et al., 2001). Therefore, we referred to a report in which B7-1/B7-2/CTLA-4^−/−^ mice were generated, and they were found to have a normal life span and produce CTLA-4–deficient T cells (Greenwald et al., 2002). We investigated the role of CTLA-4 in the genotoxic stress response induced by zeocin treatment by comparing freshly isolated CD4^+^ T cells from B7-1/B7-2^−/−^ and B7-1/B7-2/CTLA-4^−/−^ mice. We evaluated the effects of the zeocin dose on cell apoptosis. B7-1/B7-2^−/−^ and B7-1/B7-2/CTLA-4^−/−^ cells were pretreated with increasing concentrations of zeocin (0, 100, 200, 500, and 800 μg/ml) for 1 h and then cultured in fresh media without zeocin for 24 h. In the absence of zeocin (ctl) and in the presence of low zeocin doses (100 and 200 μg/ml), the B7-1/B7-2^−/−^ and B7-1/B7-2/CTLA-4^−/−^ cells exhibited similar levels of apoptosis. Treatment with 500 μg/ml zeocin resulted in an obvious increase in the B7-1/B7-2^−/−^ cell apoptosis rate compared with the B7-1/B7-2/CTLA-4^−/−^ cell apoptosis rate. These cells showed approximately equal apoptosis rates after treatment with 800 μg/ml zeocin (Figure 1A). Therefore, we chose to administer a 500 μg/ml zeocin pulse treatment to detect the apoptosis rates at different times (0, 4, 8, 12, and 24 h). The results showed gradual increases in the apoptosis rates at 0 and 4 h after a 1-h transient treatment with 500 μg/ml zeocin, with the B7-1/B7-2^−/−^ cells exhibiting a rate similar to that of the B7-1/B7-2/CTLA-4^−/−^ cells. After zeocin treatment for 8, 12, and 24 h, the B7-1/B7-2^−/−^ cells exhibited a significantly higher apoptosis rate (approximately twofold greater) than the B7-1/B7-2/CTLA-4^−/−^ cells (Figures 1B,C). Western blotting analyses were performed to examine the levels of apoptosis markers (cleaved caspase-3 and cleaved PARP) and confirm the apoptosis rates, and the results showed marked increases in the levels of these proteins in the B7-1/B7-2^−/−^ cells after zeocin treatment for 12 and 24 h (Figure 1D), indicating that the B7-1/B7-2^−/−^ cells are hypersensitive to DSBs induced by zeocin. In other words, CTLA-4 induced massive cell apoptosis after DNA damage induction.

### Zeocin Treatment Causes Overwhelming DNA Damage in CTLA-4–Expressing Cells

**FIGURE 2:**
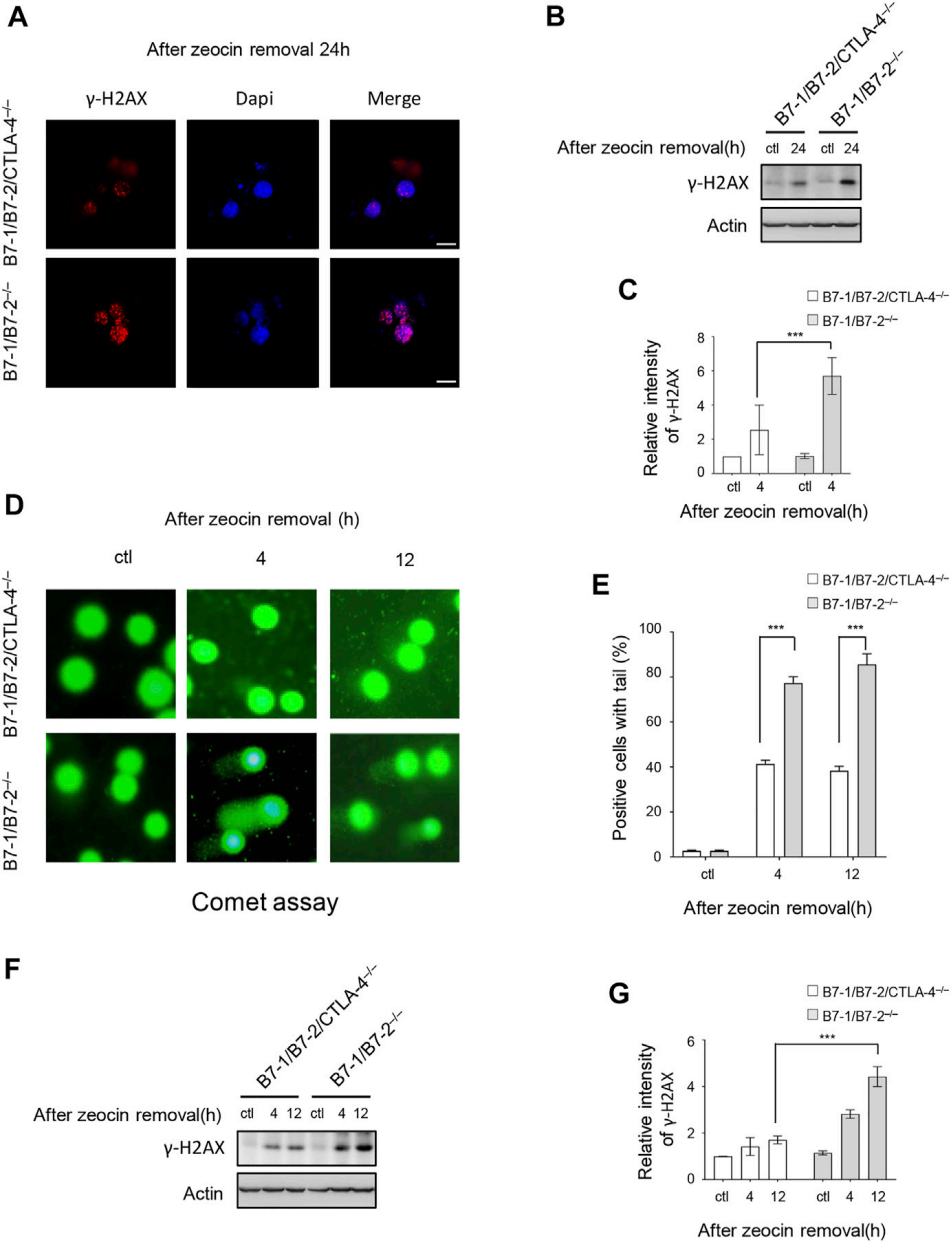
Zeocin treatment causes overwhelming DNA damage in CTLA-4–expressing cells. CD4^+^ T cells from B7-1/B7-2^−/−^ and B7-1/B7-2/CTLA-4^−/−^ mice were treated with 500 μg/ml zeocin for 1 h and then released and harvested at the indicated times. **(A)** Representative bright field and immunofluorescence images of γH2AX in treated T cells. Scale bar, 10 μm. **(B)** Cells prepared for the assay shown in panel A were analyzed for γH2AX levels using western blotting. **(C)** Quantification of the γH2AX immunoblot intensity. Each experiment was independently performed three times (*n* = 3). **(D)** DNA damage was assessed using the comet assay. Representative images of cellular DNA damage are presented. **(E)** Histogram showing the percentage of T cells with tail DNA greater than 10%. Experiments were performed three times independently (*n* = 3), and more than 100 cells per sample were counted in each experiment. Data are presented as the mean ±SEM; ***p < 0.001. **(F)** DNA damage was determined by measuring changes in the γH2AX levels in the cells shown in panel C using western blotting. **(G)** Quantification of the γH2AX band intensity in the corresponding groups. Each experiment was independently performed three times (*n* = 3).

DNA-damaging agents are potent inducers of cell apoptosis (Roos and Kaina, 2013). We tested whether CTLA-4 induced more DNA damage after drug treatment to investigate the role of CTLA-4 in apoptosis triggered by DNA damage. γH2AX, which refers to histone H2AX phosphorylated at Ser139, is a key protein that is involved in the cellular response to DNA damage and is a sensitive biomarker of DSBs (Rothkamm et al., 2015). Specifically, γH2AX forms nuclear foci that enable visualization and quantification of a DSB site. During apoptosis, the nuclei shrink and are fragmented, and the chromatin is increasingly condensed. Our results showed that the B7-1/B7-2^−/−^ and B7-1/B7-2/CTLA-4^−/−^ cells exhibited a typical apoptotic appearance after zeocin treatment for 24 h. We examined the level of γH2AX in apoptotic cells using immunocytochemical staining and western blotting analyses. A significantly greater amount of γH2AX was present in the B7-1/B7-2^−/−^ cells than in the B7-1/B7-2/CTLA-4−/− cells 24 h after transient zeocin treatment. Thus, H2AX is phosphorylated when cells undergo apoptosis and the B7-1/B7-2^−/−^ cells undergo excessive DNA damage during apoptosis (Figures 2A–C). Collectively, our results indicate that CTLA-4 leads to an increased DNA damage–induced apoptosis rate after drug treatment.

We confirmed the role of CTLA-4 in DNA damage by performing a DNA damage analysis at the other treatment time points to determine CTLA-4 function in the first 24 h of treatment. After the cessation of zeocin treatment and culture in a fresh medium, we chose two time points: a massive number of cells incubated with zeocin for 4 h showed imminent apoptosis, and 12 h after treatment, the apoptosis rate of the B7-1/B7-2^−/−^ cells was higher than that of the B7-1/B7-2/CTLA-4^−/−^ cells. A comet assay was performed to detect DNA damage in individual cells. As expected, zeocin treatment contributed to DNA damage, resulting in the fragmentation of DNA that produced comet tails in the assay. In the comet assay, the percentage of cells with tails (>10% with a tail DNA signal) was the determinant of DNA damage. Untreated cells did not show DNA damage; however, following transient zeocin treatment, the B7-1/B7-2^−/−^ cells displayed obvious DNA tails, and a greater number of the cells exhibited tails, indicating a large number of severe DNA breaks (Figures 2D,E). The γH2AX level in the cell sample with which the comet assay was performed was determined using immunoblotting (Figures 2F,G). Similar results were obtained, where upon zeocin pulse treatment, the B7-1/B7-2^−/−^ cells showed higher levels of γH2AX than the B7-1/B7-2/CTLA-4^−/−^ cells.

**FIGURE 3:**
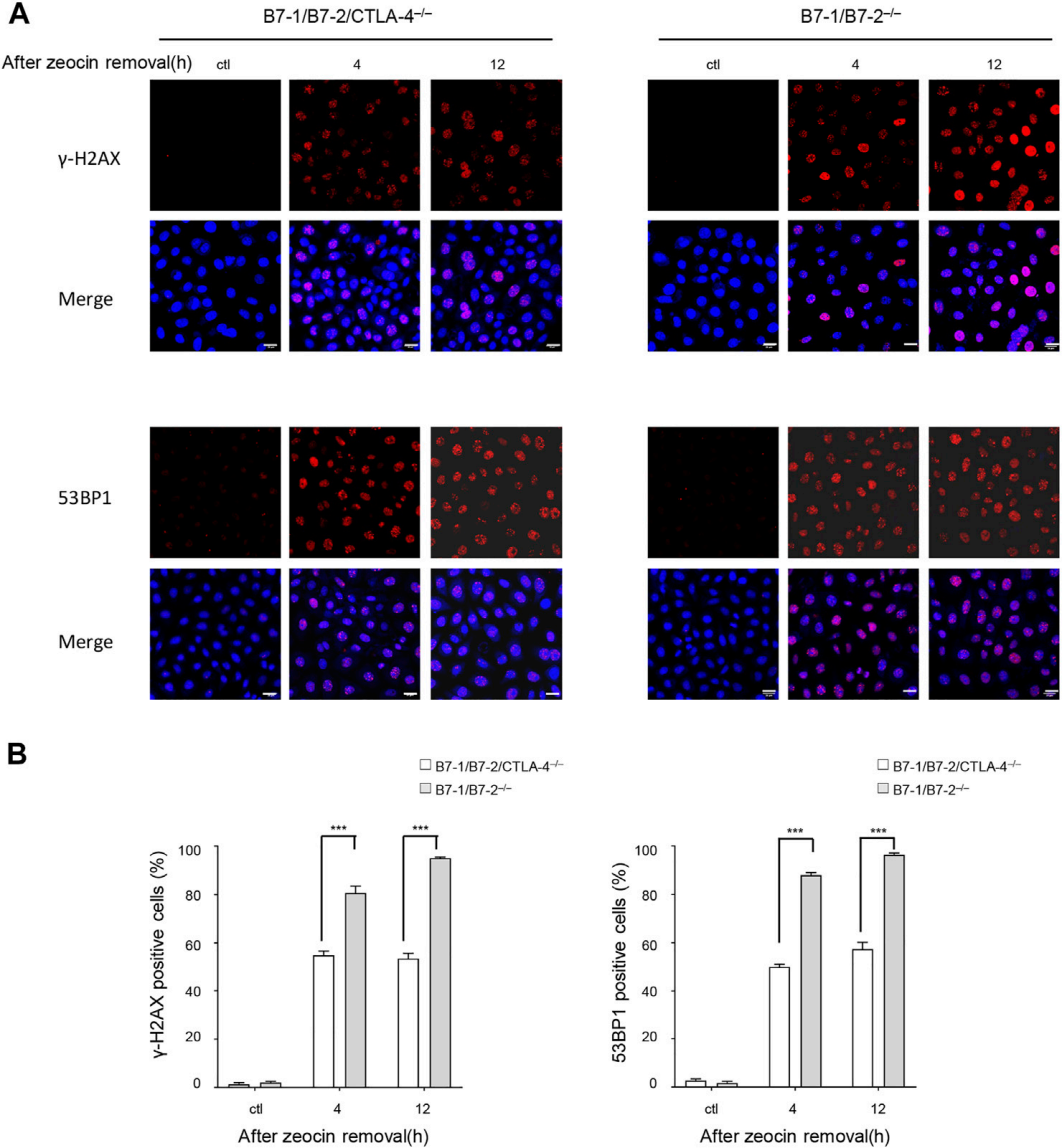
Zeocin treatment induced more γH2AX foci and 53BP1 foci in CTLA-4–expressing cells. γH2AX and 53BP1 protein accumulation at DNA damage sites forms cytologically discernible foci. **(A)** DNA damage was measured by determining the number of γH2AX and 53BP1 foci using immunofluorescence detection. Representative images are presented. The B7-1/B7-2^−/−^ and B7-1/B7-2/CTLA-4^−/−^ T cells were treated without or with 500 μg/ml zeocin for 1 h followed by culture in a normal medium for the indicated times and then stained for γH2AX (red), 53BP1 (red), and nuclei (DAPI, blue). Scale bar, 20 μm. **(B)** Quantitation of the data shown in **(A)**. The histograms represent the percentage of cells with more than 10 γH2AX and 53BP1 nuclear foci. Each experiment was independently performed three times (*n* = 3), and more than 100 cells were counted in each experiment. The data are presented as the mean ±SEM; ***p < 0.001.

The 53BP1 protein rapidly accumulates at the site of DNA damage and recruits DDR proteins to DSBs. A 53BP1 foci assay was performed to detect DSBs, as previously described (Wang et al., 2016; von Morgen et al., 2018). We examined the formation of γH2AX or 53BP1 foci and counted the number of positive cells. The extent of γH2AX and 53BP1 accumulation caused by different zeocin pulse regimens was uniformly increased in the presence of CTLA-4 (Figures 3A,B). In addition, the immunofluorescence results were consistent with the findings of the western blotting experiments and comet assays, suggesting that the B7-1/B7-2^−/−^ cells incurred a greater number of severe DSBs than the B7-1/B7-2/CTLA-4^−/−^ cells.

### CTLA-4 Affects ATM Signaling and Arrests Cells in G2/M Phase Upon DNA Damage

**FIGURE 4:**
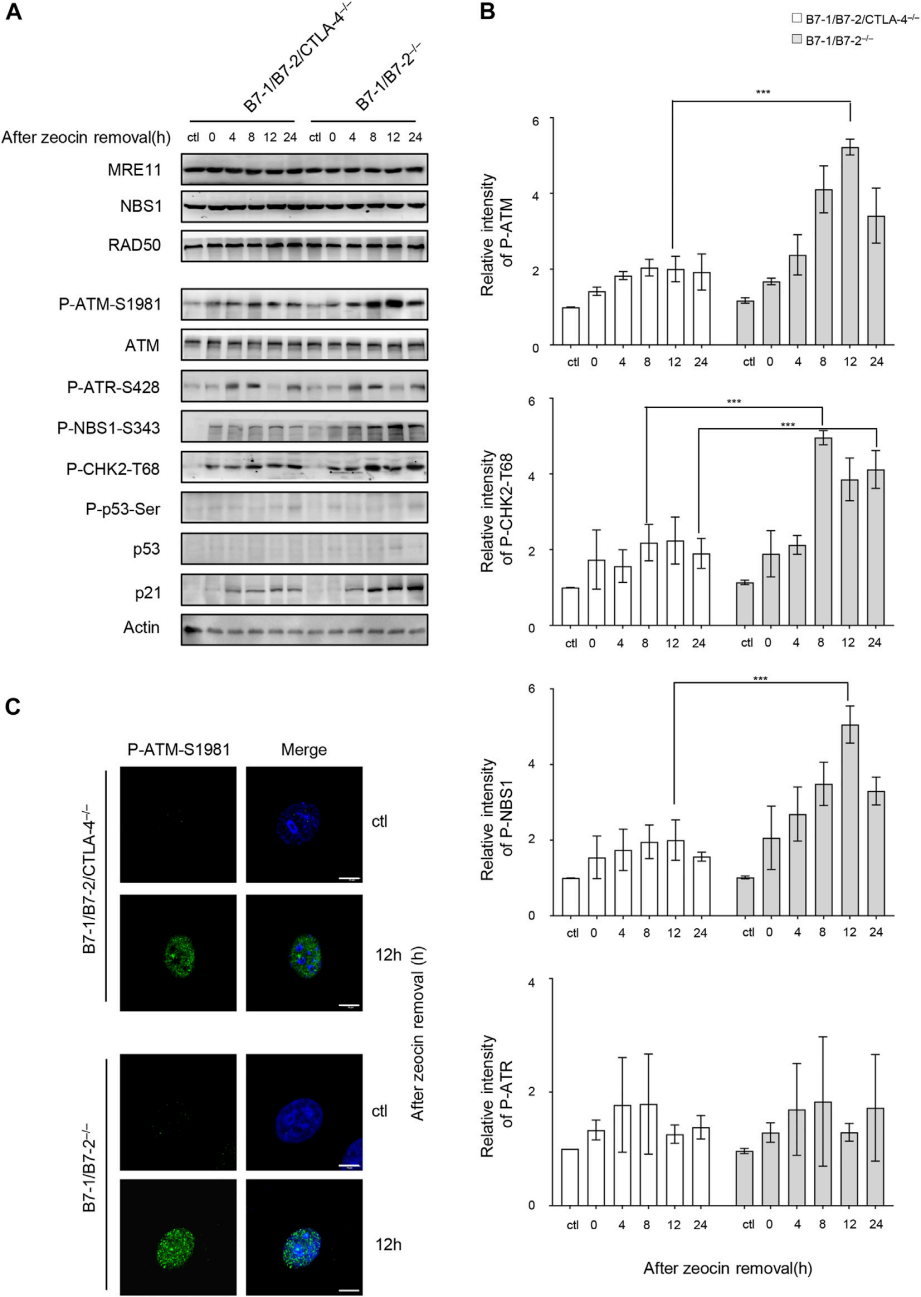
CTLA-4 causes greater zeocin-induced activation of ATM and ATM-dependent signaling pathways. The CD4^+^ T cells from B7-1/B7-2^−/−^ and B7-1/B7-2/CTLA-4^−/−^ mice were treated with 500 μg/ml zeocin for 1 h and then harvested at the indicated times. **(A)** Representative western blotting analyses are shown. The cells were lysed and analyzed at the indicated time intervals. The bar chart represents the intensity of the immunoblot bands in the corresponding group. **(B)** Quantification of P-ATM and P-CHK2-T68 immunoblots in the corresponding groups. Each experiment was independently performed three times (*n* = 3). **(C)** P-ATM-S1981 was detected using immunofluorescence staining. The cells were subjected to immunofluorescence staining using specific antibodies against P-ATM-S1981 (green) and nuclei (DAPI, blue). Scale bar, 10 μm.

We investigated whether CTLA-4 plays a role in the DDR signaling pathway to understand the mechanisms underlying the increased DNA damage in the B7-1/B7-2^−/−^ cells. ATM and ATR (ATM- and Rad3-related) proteins are key regulators and sensors of the DDR that induce apoptosis (Blackford and Jackson, 2017). The ATR signaling is activated by stalled replication forks and single-strand (ss) DNA formation during DNA replication (Awasthi et al., 2016). Our time course analysis of both the B7-1/B7-2/CTLA-4^−/−^ and B7-1/B7-2^−/−^ cells indicated that ATR activation was not obviously affected by changes in CTLA-4 expression (Figures 4A,B). ATM is activated by autophosphorylation at Ser1981 in response to the cellular DSBs; therefore, phosphorylation at Ser1981 is a marker of ATM activation. The DSBs are sensed by the MRE11, RAD50, and NBS1 (MRN) complex, which recruits ATM and plays a pivotal role in the activation of ATM signaling. ATM subsequently phosphorylates numerous targets, including the MRN complex and other downstream substrates, such as CHK2 (Bekker-Jensen et al., 2006; Zhang et al., 2012; Tripathi et al., 2018). We investigated whether CTLA-4 triggers the DDR pathway by activating ATM in cells after zeocin treatment. We analyzed ATM signaling during cellular responses to DNA damage using WB and found that the levels of ATM phosphorylation at Ser1981, CHK2 at Thr68, and NBS1 at Ser343 mediated by CTLA-4 were significantly higher in the B7-1/B7-2^−/−^ cells than in the B7-1/B7-2/CTLA-4^−/−^ cells. In addition, the activation of p53 has been previously shown to be critical for determining the cell fate after DNA is damaged (Zager and Johnson, 2019). The levels of p53 and p53 phosphorylated at Ser15 were negligibly detected and were accompanied by an increase in the expression of the target protein p21 in the B7-1/B7-2^−/−^ and B7-1/B7-2/CTLA-4^−/−^ cells treated with zeocin; p21 expression was more pronounced in the B7-1/B7-2^−/−^ cells (Figure 4A). We then examined ATM activation in these cells after zeocin treatment for 8 h using immunofluorescence staining with antibody probes specific for phosphorylated ATM. Consistent with the immunoblot data, zeocin-induced phosphorylation of ATM was more significant in the nucleus of the B7-1/B7-2^−/−^ cells than that of the B7-1/B7-2/CTLA-4^−/−^ cells (Figure 4C). We concluded from these results that CTLA-4 leads to increased phosphorylation of ATM kinase in zeocin-treated cells and thus induces DDR.

**FIGURE 5:**
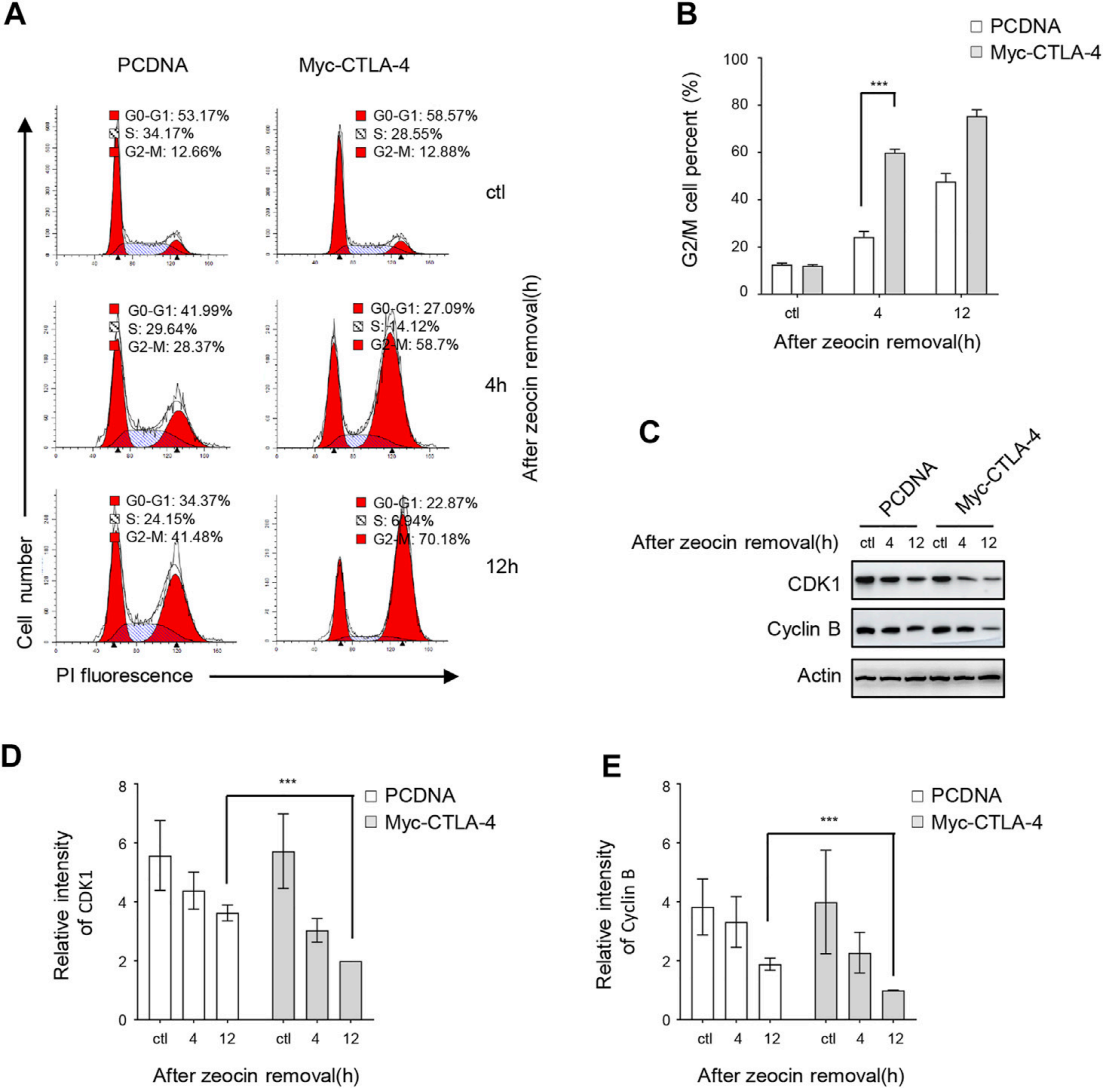
CTLA-4 promotes zeocin-induced cell cycle arrest in the G2/M phase. HeLa cells were transfected with pcDNA and Myc-CTLA-4. After 24 h, the cells were exposed to 500 μg/ml zeocin for 1 h and harvested at the indicated times. **(A)** The cell cycle distribution was analyzed using flow cytometry. **(B)** The percentages of cells in the G2/M cycle phase are depicted. **(C)** Levels of the cell cycle regulatory proteins CDK1 and cyclin B were detected using western blotting. **(D,E)** Quantification of immunoblots of CDK1 and cyclin B levels in the corresponding groups.

The CD4^+^ T lymphocytes are difficult to maintain and remodel to some extent; therefore, we designed a feasible model system in which CTLA-4 was expressed at various levels. Furthermore, we characterized CTLA-4 involvement in the DDR using a model of transiently transfected HeLa cells expressing Myc-CTLA-4 to construct a CTLA-4 overexpression system. The cellular responses to DNA damage usually include the induction of cell cycle arrest (Kramer et al., 2004; Smith et al., 2010). The nuclear DNA content of transiently transfected HeLa cells exposed to zeocin was detected using flow cytometry analysis to determine the regulatory effect of CTLA-4 on the cell cycle. In the untreated control group, CTLA-4 overexpression did not affect the cell cycle (Figures 5A,B). However, the cell cycle analysis showed that zeocin treatment increased the number of pcDNA-transfected cells and CTLA-4–transfected cells in the G2/M phase (Figures 5A,B), consistent with previous findings (Tounekti et al., 1993). Moreover, the number of CTLA-4 transfected in the G2/M phase was significantly increased after zeocin treatment. For confirmation, the expression of the G2/M cell cycle–regulated proteins [cyclin-dependent protein kinase 1 (CDK1) and cyclin B] was determined using WB. In cells overexpressing CTLA-4, the levels of CDK1 and cyclin B were elevated compared with those in cells expressing pcDNA (Figures 5C–E). Consistent with the flow cytometry results, CTLA-4 caused G2/M arrest of cells treated with zeocin.

### CTLA-4 Interacts With PP2A, Promotes the Translocation of PP2A Into the Cytoplasm, and Induces ATM Activation After Zeocin Treatment

**FIGURE 6:**
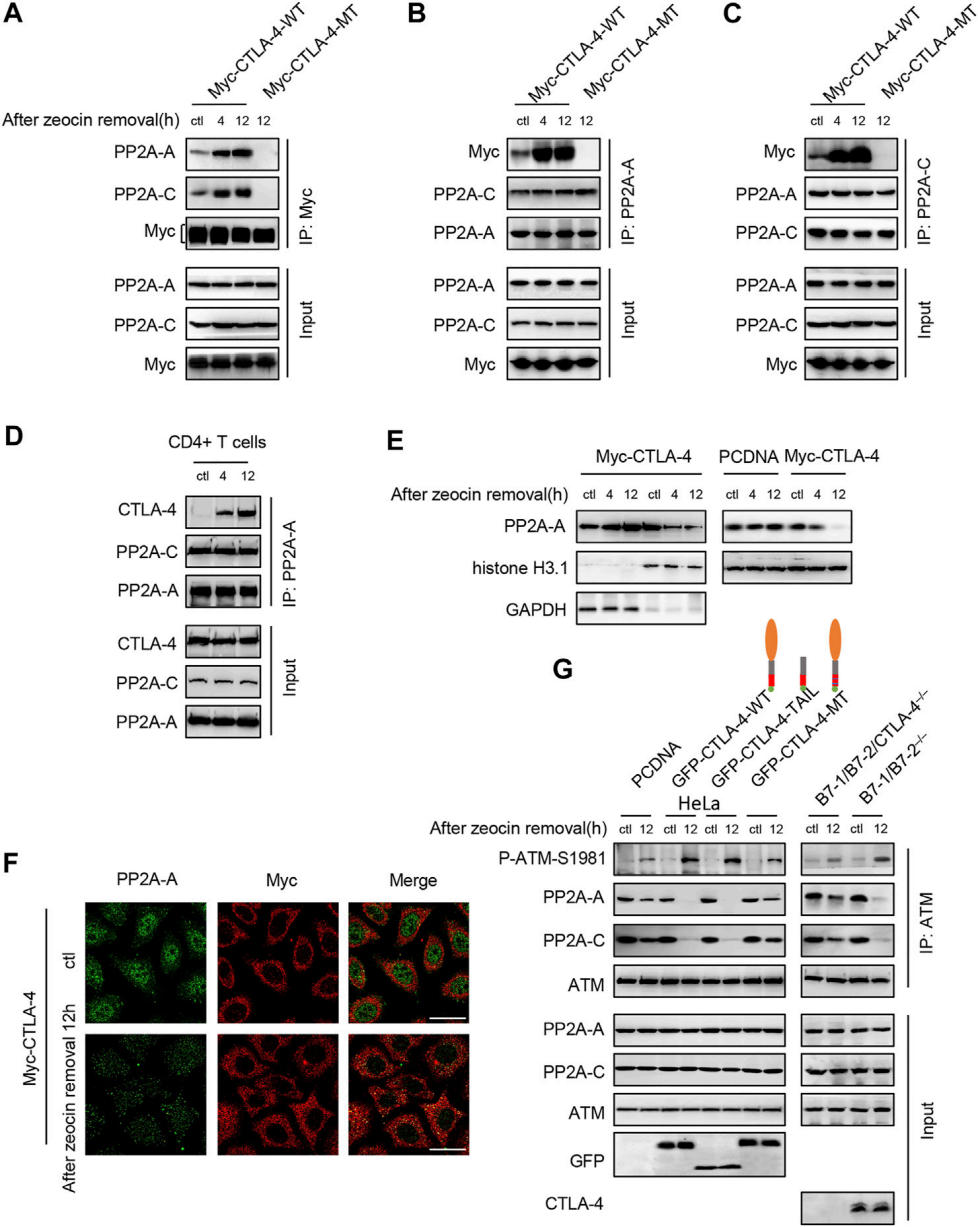
CTLA-4 interacts with PP2A, promotes the translocation of PP2A-A into the cytoplasm, and induces autophosphorylation of ATM at Ser1981 after zeocin treatment. **(A–C)** Co-IP analysis of the interaction of CTLA-4 with PP2A. HeLa cells were transfected with Myc-CTLA-4-WT and Myc-CTLA-4-MT proteins with the Y201F and Y218F mutations. About 24 h after transfection, the cells were treated with 500 μg/ml zeocin for 1 h and harvested at the indicated times. **(D)** For endogenous co-immunoprecipitation experiments, CD4^+^ T cells from B7-1/B7-2^−/−^ mice were lysed. **(E)** Nuclear and cytoplasmic levels of PP2A-A in HeLa cells were detected using western blotting. HeLa cells were transfected with Myc-CTLA-4 (left panel), and pcDNA and Myc-CTLA-4 (right panel) for 24 h. At the indicated times after cessation of treatment, the cells were lysed and separated into nuclear and cytosolic fractions. GAPDH, a marker of the cytosolic fraction; histone H3.1, a marker of the nuclear fraction. **(F)** Double immunofluorescence staining was performed to detect the PP2A-A and CTLA-4 proteins and their colocalization in HeLa cells. HeLa cells were transfected with Myc-CTLA-4 for 24 h. The cells were treated with 500 μg/ml zeocin for 1 h, harvested at the indicated time points, and then subjected to immunofluorescence staining using specific antibodies against PP2A-A (green), CTLA-4 (red), and Scale bar, 20 μm. **(G)** ATM was immunoprecipitated and immunoblotted to detect the levels of P-ATM-S1981, total ATM, and the PP2A-A and PP2A-C subunits. Right panel: HeLa cells were transfected with pcDNA, GFP-CTLA-4-WT, a GFP-CTLA-4-TAIL (tail only) mutant, and GFP-CTLA-4-MT sequences containing Y201F and Y218F mutations. About 24 h after transfection, the cells were treated with 500 μg/ml zeocin for 1 h and harvested at the indicated time points. The CTLA-4 extracellular domain is shown as orange ovals, the transmembrane domain is indicated by gray bars, the CTLA-4 cytoplasmic tail is shown as red bars, the double-mutant Y201F/Y218 is shown with blue lines, and GFP is indicated by green circles. Left panel: CD4^+^ T cells from B7-1/B7-2^−/−^ and B7-1/B7-2/CTLA-4^−/−^ mice were treated with 500 μg/ml zeocin for 1 h and then released and harvested at the indicated time points.

CTLA-4 mainly resides in intracellular vesicles and is rapidly transported to the cell surface only upon T-cell activation (Chikuma, 2017). Some reports have discovered that the CTLA-4 cytoplasmic domain interacts with PP2A, which involves CTLA-4 residues surrounding Y201 (Baroja et al., 2002). We tested the interaction of PP2A and CTLA-4 in the HeLa cells transfected with the wild-type CTLA-4 (WT) and mutant CTLA-4 (MT) which was mutated at tyrosine residues 201 and 218, after zeocin treatment. A co-IP assay with a Myc antibody against CTLA-4 revealed that the CTLA-4 interaction with endogenous PP2A increased after zeocin treatment (Figure 6A). Anti-PP2A-A and anti-PP2A-C antibodies also co-precipitated CTLA-4, and the amount of co-precipitated CTLA-4 was higher after the HeLa cells were treated with zeocin (Figures 6B,C). These findings were confirmed in primary murine T cells (Figure 6D). We found that mutating the conserved tyrosine residues present in the CTLA-4 tail abrogated the PP2A-CTLA-4 interaction (Figures 6A–C, lane 4). Cell lysates subjected to WB with anti-PP2A and anti-Myc showed equivalent sample loading.

PP2A interacts with ATM and inhibits ATM autophosphorylation and activity in the cell nucleus (Goodarzi et al., 2004; Freeman and Monteiro, 2010). We examined whether CTLA-4 affected the subcellular expression and localization of PP2A during DNA damage–induced stress to determine the mechanism by which CTLA-4 modulates the ATM signaling pathway. Interestingly, in zeocin-treated cells, the interaction with CTLA-4 promoted PP2A translocation from the nucleus to the cytoplasm (Figures 6E,F). We then investigated whether PP2A translocation to the cytoplasm affected ATM activation. Our results suggested that CTLA-4 contributed to the PP2A translocation to the cytoplasm, away from ATM, and induced ATM activation in cells treated with zeocin (Figure 6G, lane 4). CTLA-4 resides in the endocytic vesicles, with the tail located in the cytoplasm (Lo et al., 2015). We next wanted to explore whether the CTLA-4 tail alone was sufficient for PP2A binding. We found that the CTLA-4 cytoplasmic tail was sufficient and necessary for the co-IP of PP2A and activation of ATM after the treatment with zeocin (Figure 6G). These findings were confirmed with CD4^+^ T cells from B7-1/B7-2^−/−^ and B7-1/B7-2/CTLA-4^−/−^ mice (Figure 6G, left panel). Our results indicated that the cytoplasmic tail of CTLA-4 attaches PP2A to the cytoplasmic vesicles.

**FIGURE 7:**
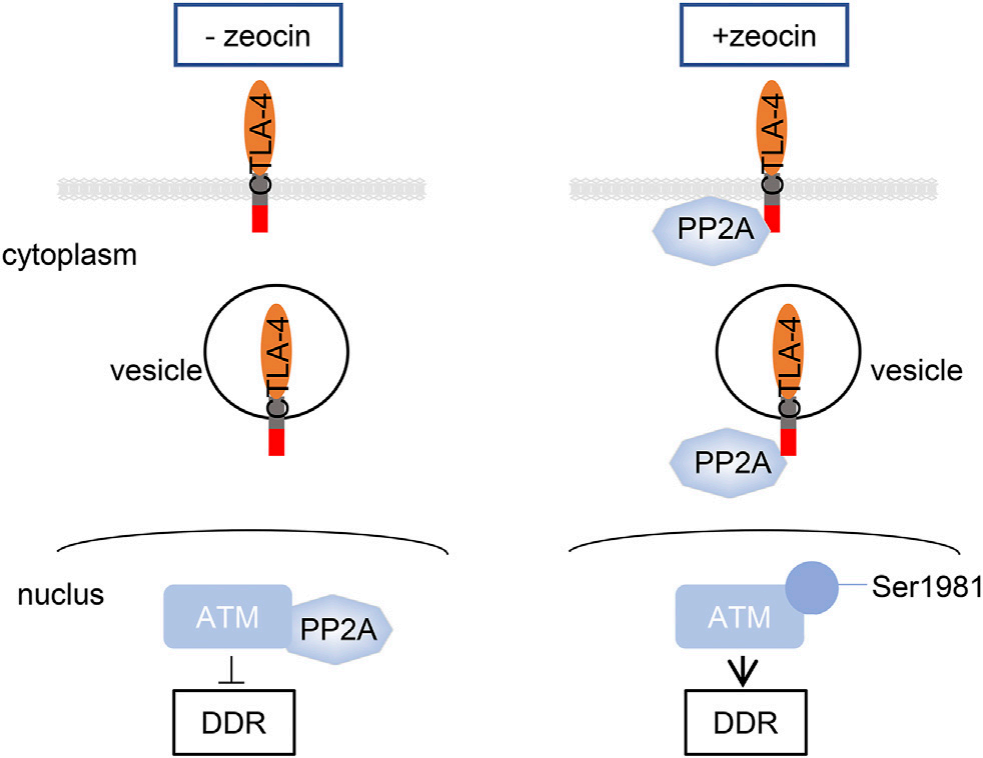
CTLA-4 activates ATM by anchoring the ATM inhibitor PP2A in the cytoplasm following transient treatment with zeocin in T cells, exacerbating the DNA damage response and inducing apoptosis.

In this study, we found that CTLA-4 activates ATM by sequestering the ATM inhibitor PP2A in the cytoplasm following transient treatment with zeocin in T cells, exaggerating the DNA damage response and inducing apoptosis (Figure 7).

## Discussion

CTLA-4 is a well-recognized immune checkpoint that can be therapeutically targeted. The most important aspect of the current understanding of CTLA-4 function is its role as an external receptor, although CTLA-4 is predominantly located intracellularly. Three compartments containing intracellular CTLA-4 have been identified: the trans-Golgi apparatus (TGN), endosomes, and lysosomes (Valk et al., 2006). The cytoplasmic tail of CTLA-4 binds to the PI3K SH2 domains SHP-2 and PP2A, leading to posttranslational modifications of downstream targets and activation of the corresponding intracellular signaling pathways (Chuang et al., 2000; Chikuma et al., 2003; Rudd et al., 2009; Iiyama et al., 2021). Recent studies have focused on the regulation of CTLA-4 subcellular localization and the capacity of CTLA-4 to reduce T-cell activation. CTLA-4 resides on tumor cells at various densities and triggers apoptosis associated with the sequential activation of caspase-8 and caspase-3 (Contardi et al., 2005). CTLA-4 arrests cell cycle progression and then inhibits T-cell proliferation by suppressing IL-2 production (Yakoub and Schulke, 2019). CTLA-4 may also induce Fas cell surface death receptor–independent apoptosis of activated T cells (Sun et al., 2008). Previously, CTLA-4 was suggested to exert these regulatory functions in a ligand interaction-dependent manner (Contardi et al., 2005). In our study, we found that CTLA-4 interacts with PP2A to activate DNA damage–induced apoptosis.

In our system, a large number of γH2AX foci were detected in cells treated with 500 μg/ml zeocin for 24 h by using confocal microscopy (Figure 2A). These data support the results reported by Mukherjee et al. (2006), who indicated that H2AX is phosphorylated during DNA fragmentation and is related to apoptosis. In addition, the increase in the γH2AX foci depends on ATM kinase phosphorylation and activation (Banath et al., 2004). Therefore, we examined the activation of the ATM signaling pathway. In the cells treated with zeocin, the phosphorylation of ATM, CHK2, and NBS1 showed a clear increasing trend (Figures 4A,B). This finding is consistent with those of previous studies showing that ATM plays a pivotal role in sensing DNA damage in cells pretreated with DSB-inducing agents and undergoes autophosphorylation at residue S1981, resulting in an increase in the active ATM kinase levels and activation of downstream signal transduction. The MRN complex is the initial sensor of DNA DSB damage. The levels of the MRN complex did not increase with zeocin treatment, consistent with the results reported by Zhou et al. (2017).

Generally, in the presence of a low amount of DNA damage, p53 initiates DNA damage repair, and it is continuously activated by high levels of DNA damage, which promotes apoptosis to remove irreparably damaged cells (Zhang et al., 2011; Roos et al., 2016). Consistent with the broad and sometimes contradictory functions of p53 reported in recent years, the mechanisms by which p53 is activated and stabilized and the downstream events of this activation, which exhibit cell type–specific patterns, remain unclear. Our data (Figure 4A) are consistent with previous findings indicating that the total protein and phosphorylation levels of p53 were negligible in resting CD4^+^ T cells after the treatment with zeocin, as indicated by WB (Hu et al., 2018). However, the expression of the p53 downstream target gene p21 was significantly increased in the B7-1/B7-2^−/−^ cells after zeocin treatment, which may have been due to other ATM activators inducing the expression of p21 (Ju and Muller, 2003; Ocker and Schneider-Stock, 2007). This finding is consistent with the observation that ATM functions through p53-independent signaling pathways to activate p53 downstream targets during cell growth and survival (Uhlemeyer et al., 2020).

Because DNA damage–mediated cell cycle checkpoint progression is essential for DDR and ATM function, which are primarily activated by DSB signaling that triggers cell cycle checkpoint activation, we examined the effect of CTLA-4 on cell cycle progression. The CDK1/cyclin B complex plays an important role in regulating the G2/M phase and facilitates the G2/M cell cycle transition (McGrath et al., 2005). The level of the CDK1/cyclin B complex has been shown to be reduced during cell cycle arrest in the G2/M phase (Gavet and Pines, 2010). In the present study, the cell cycle distribution was analyzed using flow cytometry and quantification of the DNA content (Figure 5A), and CTLA-4 was attributed to a G2/M phase delay in the cell cycle; this finding was supported by WB showing a decrease in cyclin B and CDK1 levels in CTLA-4–overexpressing cells after zeocin treatment (Figures 5C–E). The results of these two experiments are summarized as follows: following genomic stress induced by zeocin treatment, CTLA-4–expressing cells showed a profound delay in the G2/M phase transition. The cells were arrested in the G2/M phase upon activation of the ATM/CHK2/Cdc25 pathway through a mechanism independent of p53 (Tsou et al., 2006; Chang et al., 2016). This outcome may be at least partially explained by ATM activation and CTLA-4–induced arrest of cells in the G2/M phase following DNA damage.

CTLA-4 interaction with PP2A has been reported to be critical for the regulatory activity of T cells mediated by the PI3K-PKB-AKT pathway (Baroja et al., 2002; Resjo et al., 2002). In our study, CTLA-4 interacted with PP2A under resting conditions in normal cells (Figures 6A–D, line 1), consistent with the study by Baroja et al. (2002). According to the study by Goodarzi et al. (2004), the binding of PP2A to ATM in the cell nucleus tends to suppress ATM activation. Under normal circumstances without DNA damage, ATM constitutively interacts with PP2A. In response to DNA damage, which releases the inhibition by PP2A, ATM activation is increased (Volonte et al., 2009). This supposition appears to be consistent with our results (Figure 6G). Interestingly, in our system, a majority of PP2A colocalized with CTLA-4 in the cytoplasm, not near ATM (Figure 6F), after critical DNA-damaging stress induction. This colocalization excessively activated ATM autophosphorylation from a basal state, exaggerated the response to DNA damage, and induced cellular apoptosis. Furthermore, the tail of CTLA-4 was sufficient for PP2A binding and activation of ATM. This result is consistent with previous studies by Chuang et al. (2000). Our data suggested that CTLA-4 triggers a signaling network that culminates in an increase in ATM function in response to DNA damage. These observations present a new and promising approach to understanding cancer and developing new molecular therapeutics. Further *in vivo* evaluation is needed to validate the function of CTLA-4.

PP2A is associated with the cytoplasmic domains of CD28 (Chuang et al., 2000). However, this association invoked a DNA damage response in cells treated with zeocin (data not shown). By analyzing the structure of the cytoplasmic domains with coiled-coil proteins or by altering the phosphorylation state of tyrosine residues, a research group (Chuang et al., 2000) observed the difference between the mechanisms of CTLA-4 and PP2A interaction or the difference in affinity of CTLA-4 for PP2A and CD28, ultimately showing that CD28 interacts with PP2A. Thus, a different binding mechanism may account for the inability of CD28 to interact with PP2A in response to DNA damage.

Our model is supported by the findings of Lingel et al. (2017), to some extent. Other groups identified some downstream effectors of CTLA-4, including Ku70, BRCA1, SSRP1, and WRNIP1, as well as the associated signaling pathways in T cells, showing that these molecules play fundamental roles in the DDR (Kumari et al., 2009; Saha and Davis, 2016; Yoshimura et al., 2017; Yue et al., 2020). Therefore, these effectors should be studied to determine the numerous molecular mechanisms of the CTLA-4–mediated DDR. Furthermore, CTLA-4 is involved in regulating cell cycle progression and cell proliferation (Greenwald et al., 2002). However, further studies are required to investigate the mechanism by which CTLA-4 senses DNA damage.

What is the clinical significance of CTLA-4–mediated apoptosis induced by DNA damage in T cells? Previous studies have shown that chemotherapy or radiotherapy elicits antitumor effects through the immune system, which might lead to increased immunotherapy activity and better outcomes. Immunotherapy combined with chemoradiotherapy may result in an enhanced curative effect on cancer (Goyal et al., 2015; Chicas-Sett et al., 2018; Paz-Ares et al., 2021). Our study suggested that T cells may maintain their immune capacity under high-stress conditions by inhibiting CTLA-4–mediated apoptosis, which helps us to understand that CTLA-4 inhibitors such as ipilimumab function not only as terminators of CTLA-4 immune checkpoint signaling but also as coordinators for maintaining the immune capacity of T cells, which is clinically important for patients with tumors who are receiving immunotherapy combined with conventional cancer therapies.

Of course, our study has some limitations. Most CTLA-4 molecules reside in the cytoplasmic vesicles, and only a fraction transiently resides on the T-cell surface. Consistent with our conclusion, PP2A is adsorbed onto the vesicle surface by CTLA-4. Colocalization of the recycling endosome marker Rab11, TGN marker STX6, and the PP2A protein in T cells with DNA damage should be examined using immunofluorescence analyses to test this hypothesis. Moreover, the extracellular segment of CTLA-4 binds to B7 ligands with high affinity. We did not determine whether this binding alters the interaction of the CTLA-4 cytoplasmic tail with PP2A in cells with DNA damage in our study. Eventually, the findings of this study must be validated *in vivo*.

In the present study, we determined that CTLA-4 plays an essential role in apoptosis induced by DNA damage. This finding will help to partially explain the reasons why immunotherapy-based strategies combined with chemotherapy and/or radiotherapy exhibit greater therapeutic efficacy.

## Data Availability

The original contributions presented in the study are included in the article/Supplementary Material, further inquiries can be directed to the corresponding author.

## References

[B1] Awasthi P. Foiani M. Kumar A. (2016). ATM and ATR Signaling at a Glance. J. Cel Sci 129 (6), 1285. 10.1242/jcs.188631 26979625

[B2] Banáth J. P. Macphail S. H. Olive P. L. (2004). Radiation Sensitivity, H2AX Phosphorylation, and Kinetics of Repair of DNA Strand Breaks in Irradiated Cervical Cancer Cell Lines. Cancer Res. 64 (19), 7144–7149. 10.1158/0008-5472.CAN-04-1433 15466212

[B3] Baroja M. L. Vijayakrishnan L. Bettelli E. Darlington P. J. Chau T. A. Ling V. (2002). Inhibition of CTLA-4 Function by the Regulatory Subunit of Serine/threonine Phosphatase 2A. J. Immunol. 168 (10), 5070–5078. 10.4049/jimmunol.168.10.5070 11994459

[B4] Bekker-Jensen S. Lukas C. Kitagawa R. Melander F. Kastan M. B. Bartek J. (2006). Spatial Organization of the Mammalian Genome Surveillance Machinery in Response to DNA Strand Breaks. J. Cel Biol 173 (2), 195–206. 10.1083/jcb.200510130 PMC2063811 16618811

[B5] Blackford A. N. Jackson S. P. (2017). ATM, ATR, and DNA-PK: The Trinity at the Heart of the DNA Damage Response. Mol. Cel 66 (6), 801–817. 10.1016/j.molcel.2017.05.015 28622525

[B6] Bouwman P. Jonkers J. (2012). The Effects of Deregulated DNA Damage Signalling on Cancer Chemotherapy Response and Resistance. Nat. Rev. Cancer 12 (9), 587–598. 10.1038/nrc3342 22918414

[B7] Chambers C. A. Kuhns M. S. Egen J. G. Allison J. P. (2001). CTLA-4-mediated Inhibition in Regulation of T Cell Responses: Mechanisms and Manipulation in Tumor Immunotherapy. Annu. Rev. Immunol. 19, 565–594. 10.1146/annurev.immunol.19.1.565 11244047

[B8] Chang M.-C. Chen Y.-J. Liou E. J.-W. Tseng W.-Y. Chan C.-P. Lin H.-J. (2016). 7-Ketocholesterol Induces ATM/ATR, Chk1/Chk2, PI3K/Akt Signalings, Cytotoxicity and IL-8 Production in Endothelial Cells. Oncotarget 7 (46), 74473–74483. 10.18632/oncotarget.12578 27740938 PMC5342680

[B9] Chang X. Lu X. Guo J. Teng G.-J. (2019). Interventional Therapy Combined with Immune Checkpoint Inhibitors: Emerging Opportunities for Cancer Treatment in the Era of Immunotherapy. Cancer Treat. Rev. 74, 49–60. 10.1016/j.ctrv.2018.08.006 30831375

[B10] Chicas-Sett R. Morales-Orue I. Rodriguez-Abreu D. Lara-Jimenez P. (2018). Combining Radiotherapy and Ipilimumab Induces Clinically Relevant Radiation-Induced Abscopal Effects in Metastatic Melanoma Patients: A Systematic Review. Clin. Translational Radiat. Oncol. 9, 5–11. 10.1016/j.ctro.2017.12.004 PMC5862682 29594244

[B11] Chikuma S. (2017). CTLA-4, an Essential Immune-Checkpoint for T-Cell Activation. Curr. Top. Microbiol. Immunol. 410, 99–126. 10.1007/82_2017_61 28900679

[B12] Chikuma S. Imboden J. B. Bluestone J. A. (2003). Negative Regulation of T Cell Receptor-Lipid Raft Interaction by Cytotoxic T Lymphocyte-Associated Antigen 4. J. Exp. Med. 197 (1), 129–135. 10.1084/jem.20021646 12515820 PMC2193802

[B13] Chuang E. Fisher T. S. Morgan R. W. Robbins M. D. Duerr J. M. Vander Heiden M. G. (2000). The CD28 and CTLA-4 Receptors Associate with the Serine/threonine Phosphatase PP2A. Immunity 13 (3), 313–322. 10.1016/s1074-7613(00)00031-5 11021529

[B14] Ciccia A. Elledge S. J. (2010). The DNA Damage Response: Making it Safe to Play with Knives. Mol. Cel 40 (2), 179–204. 10.1016/j.molcel.2010.09.019 PMC2988877 20965415

[B15] Contardi E. Palmisano G. L. Tazzari P. L. Martelli A. M. Falà F. Fabbi M. (2005). CTLA-4 Is Constitutively Expressed on Tumor Cells and Can Trigger Apoptosis upon Ligand Interaction. Int. J. Cancer 117 (4), 538–550. 10.1002/ijc.21155 15912538

[B16] Finn K. Lowndes N. F. Grenon M. (2012). Eukaryotic DNA Damage Checkpoint Activation in Response to Double-Strand Breaks. Cell. Mol. Life Sci. 69 (9), 1447–1473. 10.1007/s00018-011-0875-3 22083606 PMC11115150

[B17] Freeman A. K. Monteiro A. N. (2010). Phosphatases in the Cellular Response to DNA Damage. Cell Commun. Signaling 8, 27. 10.1186/1478-811X-8-27 PMC2954851 20860841

[B18] Gavet O. Pines J. (2010). Progressive Activation of CyclinB1-Cdk1 Coordinates Entry to Mitosis. Dev. Cel 18 (4), 533–543. 10.1016/j.devcel.2010.02.013 PMC3325599 20412769

[B19] Ghaderi A. (2011). CTLA4 Gene Variants in Autoimmunity and Cancer: a Comparative Review. Iran J. Immunol. 8 (3), 127–149. 21931200 10.22034/iji.2011.17019

[B20] Goodarzi A. A. Jonnalagadda J. C. Douglas P. Young D. Ye R. Moorhead G. B. G. (2004). Autophosphorylation of Ataxia-Telangiectasia Mutated Is Regulated by Protein Phosphatase 2A. EMBO J. 23 (22), 4451–4461. 10.1038/sj.emboj.7600455 15510216 PMC526470

[B21] Goyal S. Silk A. W. Tian S. Mehnert J. Danish S. Ranjan S. (2015). Clinical Management of Multiple Melanoma Brain Metastases: A Systematic Review. JAMA Oncol. 1 (5), 668–676. 10.1001/jamaoncol.2015.1206 26181286 PMC5726801

[B22] Greenwald R. J. Oosterwegel M. A. van der Woude D. Kubal A. Mandelbrot D. A. Boussiotis V. A. (2002). CTLA-4 Regulates Cell Cycle Progression during a Primary Immune Response. Eur. J. Immunol. 32 (2), 366–373. 10.1002/1521-4141(200202)32:2<366:aid-immu366>3.0.co;2-5 11807776

[B23] Haanen J. B. A. G. Robert C. (2015). Immune Checkpoint Inhibitors. Prog. Tumor Res. 42, 55–66. 10.1159/000437178 26382943

[B24] Hanahan D. Weinberg R. A. (2011). Hallmarks of Cancer: the Next Generation. Cell 144 (5), 646–674. 10.1016/j.cell.2011.02.013 21376230

[B25] Hu Q. Xie Y. Ge Y. Nie X. Tao J. Zhao Y. (2018). Resting T Cells Are Hypersensitive to DNA Damage Due to Defective DNA Repair Pathway. Cell Death Dis 9 (6), 662. 10.1038/s41419-018-0649-z 29855463 PMC5981309

[B26] Iiyama M. Numoto N. Ogawa S. Kuroda M. Morii H. Abe R. (2021). Molecular Interactions of the CTLA-4 Cytoplasmic Region with the Phosphoinositide 3-kinase SH2 Domains. Mol. Immunol. 131, 51–59. 10.1016/j.molimm.2020.12.002 33386150

[B27] Janssens V. Goris J. (2001). Protein Phosphatase 2A: a Highly Regulated Family of Serine/threonine Phosphatases Implicated in Cell Growth and Signalling. Biochem. J. 353 (Pt 3), 417–439. 10.1042/0264-6021:3530417 11171037 PMC1221586

[B28] Ju R. Muller M. T. (2003). Histone Deacetylase Inhibitors Activate p21(WAF1) Expression via ATM. Cancer Res. 63 (11), 2891–2897. 12782595

[B29] Krämer A. Lukas J. Bartek J. (2004). Checking Out the Centrosome. Cell Cycle 3 (11), 1390–1393. 10.4161/cc.3.11.1252 15483402

[B30] Kumari A. Mazina O. M. Shinde U. Mazin A. V. Lu H. (2009). A Role for SSRP1 in Recombination-Mediated DNA Damage Response. J. Cel. Biochem. 108 (2), 508–518. 10.1002/jcb.22280 PMC8215854 19639603

[B31] Lei X. Ma N. Du L. Liang Y. Zhang P. Han Y. (2020). PP2A and Tumor Radiotherapy. Hereditas 157 (1), 36. 10.1186/s41065-020-00149-7 32847617 PMC7450598

[B32] Lingel H. Wissing J. Arra A. Schanze D. Lienenklaus S. Klawonn F. (2017). CTLA-4-mediated Posttranslational Modifications Direct Cytotoxic T-Lymphocyte Differentiation. Cell Death Differ 24 (10), 1739–1749. 10.1038/cdd.2017.102 28644433 PMC5596418

[B33] Liu W. Yang Z. Chen Y. Yang H. Wan X. Zhou X. (2021). The Association between CTLA-4, CD80/86, and CD28 Gene Polymorphisms and Rheumatoid Arthritis: An Original Study and Meta-Analysis. Front. Med. 8, 598076. 10.3389/fmed.2021.598076 PMC7884472 33604347

[B34] Lo B. Zhang K. Lu W. Zheng L. Zhang Q. Kanellopoulou C. (2015). Patients with LRBA Deficiency Show CTLA4 Loss and Immune Dysregulation Responsive to Abatacept Therapy. Science 349 (6246), 436–440. 10.1126/science.aaa1663 26206937

[B35] McGrath C. F. Pattabiraman N. Kellogg G. E. Lemcke T. Kunick C. Sausville E. A. (2005). Homology Model of the CDK1/cyclin B Complex. J. Biomol. Struct. Dyn. 22 (5), 493–502. 10.1080/07391102.2005.10531227 15702922

[B36] Mukherjee B. Kessinger C. Kobayashi J. Chen B. P. C. Chen D. J. Chatterjee A. (2006). DNA-PK Phosphorylates Histone H2AX during Apoptotic DNA Fragmentation in Mammalian Cells. DNA Repair 5 (5), 575–590. 10.1016/j.dnarep.2006.01.011 16567133

[B37] Ocker M. Schneider-Stock R. (2007). Histone Deacetylase Inhibitors: Signalling towards P21cip1/waf1. Int. J. Biochem. Cel Biol. 39 (7-8), 1367–1374. 10.1016/j.biocel.2007.03.001 17412634

[B38] Opzoomer J. W. Sosnowska D. Anstee J. E. Spicer J. F. Arnold J. N. (2019). Cytotoxic Chemotherapy as an Immune Stimulus: A Molecular Perspective on Turning up the Immunological Heat on Cancer. Front. Immunol. 10, 1654. 10.3389/fimmu.2019.01654 31379850 PMC6652267

[B39] Paz-Ares L. Ciuleanu T.-E. Cobo M. Schenker M. Zurawski B. Menezes J. (2021). First-line Nivolumab Plus Ipilimumab Combined with Two Cycles of Chemotherapy in Patients with Non-small-cell Lung Cancer (CheckMate 9LA): an International, Randomised, Open-Label, Phase 3 Trial. Lancet Oncol. 22 (2), 198–211. 10.1016/S1470-2045(20)30641-0 33476593

[B40] Peres de Oliveira A. Basei F. L. Slepicka P. F. de Castro Ferezin C. Melo-Hanchuk T. D. de Souza E. E. (2020). NEK10 Interactome and Depletion Reveal New Roles in Mitochondria. Proteome Sci. 18, 4. 10.1186/s12953-020-00160-w 32368190 PMC7189645

[B41] Pistillo M. P. Tazzari P. L. Palmisano G. L. Pierri I. Bolognesi A. Ferlito F. (2003). CTLA-4 Is Not Restricted to the Lymphoid Cell Lineage and Can Function as a Target Molecule for Apoptosis Induction of Leukemic Cells. Blood 101 (1), 202–209. 10.1182/blood-2002-06-1668 12393538

[B42] Qureshi O. S. Kaur S. Hou T. Z. Jeffery L. E. Poulter N. S. Briggs Z. (2012). Constitutive Clathrin-Mediated Endocytosis of CTLA-4 Persists during T Cell Activation. J. Biol. Chem. 287 (12), 9429–9440. 10.1074/jbc.M111.304329 22262842 PMC3308817

[B43] Ramos F. Villoria M. T. Alonso-Rodríguez E. Clemente-Blanco A. (2019). Role of Protein Phosphatases PP1, PP2A, PP4 and Cdc14 in the DNA Damage Response. Cst 3 (3), 70–85. 10.15698/cst2019.03.178 PMC6551743 31225502

[B44] Resjö S. Göransson O. Härndahl L. Zolnierowicz S. Manganiello V. Degerman E. (2002). Protein Phosphatase 2A Is the Main Phosphatase Involved in the Regulation of Protein Kinase B in Rat Adipocytes. Cell Signal. 14 (3), 231–238. 10.1016/s0898-6568(01)00238-8 11812651

[B45] Roos W. P. Kaina B. (2013). DNA Damage-Induced Cell Death: from Specific DNA Lesions to the DNA Damage Response and Apoptosis. Cancer Lett. 332 (2), 237–248. 10.1016/j.canlet.2012.01.007 22261329

[B46] Roos W. P. Thomas A. D. Kaina B. (2016). DNA Damage and the Balance between Survival and Death in Cancer Biology. Nat. Rev. Cancer 16 (1), 20–33. 10.1038/nrc.2015.2 26678314

[B47] Rothkamm K. Barnard S. Moquet J. Ellender M. Rana Z. Burdak-Rothkamm S. (2015). DNA Damage Foci: Meaning and Significance. Environ. Mol. Mutagen. 56 (6), 491–504. 10.1002/em.21944 25773265

[B48] Rudd C. E. Taylor A. Schneider H. (2009). CD28 and CTLA-4 Coreceptor Expression and Signal Transduction. Immunol. Rev. 229 (1), 12–26. 10.1111/j.1600-065X.2009.00770.x 19426212 PMC4186963

[B49] Saha J. Davis A. J. (2016). Unsolved Mystery: the Role of BRCA1 in DNA End-Joining. J. Radiat. Res. 57 (Suppl. 1), i18–i24. 10.1093/jrr/rrw032 27170701 PMC4990114

[B50] Schlößer H. A. Drebber U. Kloth M. Thelen M. Rothschild S. I. Haase S. (2016). Immune Checkpoints Programmed Death 1 Ligand 1 and Cytotoxic T Lymphocyte Associated Molecule 4 in Gastric Adenocarcinoma. Oncoimmunology 5 (5), e1100789. 10.1080/2162402X.2015.1100789 27467911 PMC4910699

[B51] Shi Y. (2009). Serine/threonine Phosphatases: Mechanism through Structure. Cell 139 (3), 468–484. 10.1016/j.cell.2009.10.006 19879837

[B52] Shklovskaya E. Rizos H. (2021). MHC Class I Deficiency in Solid Tumors and Therapeutic Strategies to Overcome it. Ijms 22 (13), 6741. 10.3390/ijms22136741 34201655 PMC8268865

[B53] Smith J. Mun Tho L. Xu N. A. Gillespie D. (2010). The ATM-Chk2 and ATR-Chk1 Pathways in DNA Damage Signaling and Cancer. Adv. Cancer Res. 108, 73–112. 10.1016/B978-0-12-380888-2.00003-0 21034966

[B54] Sobhani N. Tardiel-Cyril D. R. Davtyan A. Generali D. Roudi R. Li Y. (2021). CTLA-4 in Regulatory T Cells for Cancer Immunotherapy. Cancers 13 (6), 1440. 10.3390/cancers13061440 33809974 PMC8005092

[B55] Sun T. Zhou Y. Yang M. Hu Z. Tan W. Han X. (2008). Functional Genetic Variations in Cytotoxic T-Lymphocyte Antigen 4 and Susceptibility to Multiple Types of Cancer. Cancer Res. 68 (17), 7025–7034. 10.1158/0008-5472.CAN-08-0806 18757416

[B56] Teft W. A. Chau T. A. Madrenas J. (2009). Structure-Function Analysis of the CTLA-4 Interaction with PP2A. BMC Immunol. 10, 23. 10.1186/1471-2172-10-23 19405949 PMC2683795

[B57] Teft W. A. Kirchhof M. G. Madrenas J. (2006). A Molecular Perspective of CTLA-4 Function. Annu. Rev. Immunol. 24, 65–97. 10.1146/annurev.immunol.24.021605.090535 16551244

[B58] Tounekti O. Pron G. Belehradek J. Jr. Mir L. M. (1993). Bleomycin, an Apoptosis-Mimetic Drug that Induces Two Types of Cell Death Depending on the Number of Molecules Internalized. Cancer Res. 53 (22), 5462–5469. 7693342

[B59] Tripathi V. Agarwal H. Priya S. Batra H. Modi P. Pandey M. (2018). MRN Complex-dependent Recruitment of Ubiquitylated BLM Helicase to DSBs Negatively Regulates DNA Repair Pathways. Nat. Commun. 9 (1), 1016. 10.1038/s41467-018-03393-8 29523790 PMC5844875

[B60] Tsou T.-C. Tsai F.-Y. Yeh S.-C. Chang L. W. (2006). ATM/ATR-related Checkpoint Signals Mediate Arsenite-Induced G2/M Arrest in Primary Aortic Endothelial Cells. Arch. Toxicol. 80 (12), 804–810. 10.1007/s00204-006-0110-4 16645841

[B61] Uhlemeyer C. Müller N. Griess K. Wessel C. Schlegel C. Kuboth J. (2020). ATM and P53 Differentially Regulate Pancreatic Beta Cell Survival in Ins1E Cells. PLoS One 15 (8), e0237669. 10.1371/journal.pone.0237669 32810137 PMC7437460

[B62] Valk E. Leung R. Kang H. Kaneko K. Rudd C. E. Schneider H. (2006). T Cell Receptor-Interacting Molecule Acts as a Chaperone to Modulate Surface Expression of the CTLA-4 Coreceptor. Immunity 25 (5), 807–821. 10.1016/j.immuni.2006.08.024 17070077

[B63] Van Coillie S. Wiernicki B. Xu J. (2020). Molecular and Cellular Functions of CTLA-4. Adv. Exp. Med. Biol. 1248, 7–32. 10.1007/978-981-15-3266-5_2 32185705

[B64] Vermeulen K. Berneman Z. N. Van Bockstaele D. R. (2003). Cell Cycle and Apoptosis. Cell Prolif 36 (3), 165–175. 10.1046/j.1365-2184.2003.00267.x 12814432 PMC6496173

[B65] Volonte D. Kahkonen B. Shapiro S. Di Y. Galbiati F. (2009). Caveolin-1 Expression Is Required for the Development of Pulmonary Emphysema through Activation of the ATM-P53-P21 Pathway. J. Biol. Chem. 284 (9), 5462–5466. 10.1074/jbc.C800225200 19103597 PMC2645811

[B66] von Morgen P. Lidak T. Horejsi Z. Macurek L. (2018). Nuclear Localisation of 53BP1 Is Regulated by Phosphorylation of the Nuclear Localisation Signal. Biol. Cel 110 (6), 137–146. 10.1111/boc.201700067 29603287

[B67] Wang J. Yin L. Zhang J. Zhang Y. Zhang X. Ding D. (2016). The Profiles of Gamma-H2AX along with ATM/DNA-PKcs Activation in the Lymphocytes and Granulocytes of Rat and Human Blood Exposed to Gamma Rays. Radiat. Environ. Biophys. 55 (3), 359–370. 10.1007/s00411-016-0653-6 27260225

[B68] Wang X.-B. Giscombe R. Yan Z. Heiden T. Xu D. Lefvert A. K. (2002). Expression of CTLA-4 by Human Monocytes. Scand. J. Immunol. 55 (1), 53–60. 10.1046/j.0300-9475.2001.01019.x 11841692

[B69] Wang Y. Liu Z.-G. Yuan H. Deng W. Li J. Huang Y. (2019). The Reciprocity between Radiotherapy and Cancer Immunotherapy. Clin. Cancer Res. 25 (6), 1709–1717. 10.1158/1078-0432.CCR-18-2581 30413527 PMC6420874

[B70] Yakoub A. M. Schülke S. (2019). A Model for Apoptotic-Cell-Mediated Adaptive Immune Evasion via CD80-CTLA-4 Signaling. Front. Pharmacol. 10, 562. 10.3389/fphar.2019.00562 31214024 PMC6554677

[B71] Yan Q. Zhu H. Lan L. Yi J. Yang J. (2017). Cleavage of Ku80 by Caspase-2 Promotes Non-homologous End Joining-Mediated DNA Repair. DNA Repair 60, 18–28. 10.1016/j.dnarep.2017.10.001 29065392

[B72] Yoshimura A. Seki M. Enomoto T. (2017). The Role of WRNIP1 in Genome Maintenance. Cell Cycle 16 (6), 515–521. 10.1080/15384101.2017.1282585 28118071 PMC5384577

[B73] Yue X. Bai C. Xie D. Ma T. Zhou P.-K. (2020). DNA-PKcs: A Multi-Faceted Player in DNA Damage Response. Front. Genet. 11, 607428. 10.3389/fgene.2020.607428 33424929 PMC7786053

[B74] Zager R. A. Johnson A. C. M. (2019). Acute Kidney Injury Induces Dramatic P21 Upregulation via a Novel, Glucocorticoid-Activated, Pathway. Am. J. Physiology-Renal Physiol. 316 (4), F674–F681. 10.1152/ajprenal.00571.2018 PMC6483029 30698046

[B75] Zhang T. Penicud K. Bruhn C. Loizou J. I. Kanu N. Wang Z.-Q. (2012). Competition between NBS1 and ATMIN Controls ATM Signaling Pathway Choice. Cel Rep. 2 (6), 1498–1504. 10.1016/j.celrep.2012.11.002 23219553

[B76] Zhang X.-P. Liu F. Wang W. (2011). Two-phase Dynamics of P53 in the DNA Damage Response. Proc. Natl. Acad. Sci. 108 (22), 8990–8995. 10.1073/pnas.1100600108 21576488 PMC3107314

[B77] Zhou H. Kawamura K. Yanagihara H. Kobayashi J. Zhang-Akiyama Q.-M. (2017). NBS1 Is Regulated by Two Kind of Mechanisms: ATM-dependent Complex Formation with MRE11 and RAD50, and Cell Cycle-dependent Degradation of Protein. J. Radiat. Res. 58 (4), 487–494. 10.1093/jrr/rrx014 28369484 PMC5570008

